# Bakery Waste Inclusion in the Diet of Growing Black Goat Kids: Evaluation of Performance and Health Aspects

**DOI:** 10.3390/ani15030383

**Published:** 2025-01-29

**Authors:** Belal S. Obeidat

**Affiliations:** Department of Animal Production, Faculty of Agriculture, Jordan University of Science and Technology, Irbid 22110, Jordan; bobeidat@just.edu.jo; Tel.: +962-2-7201000 (ext. 22214); Fax: +962-2-7201078

**Keywords:** bakery waste, black goat kids, blood parameters, carcass characteristics

## Abstract

The influence of bakery waste (BAWA) in the diet of black goat kids on nutrient intake, health, growth potential, and carcass features was assessed. Three treatment diets, consisting of 0 BAWA (CON), 50 g/kg BAWA (BAWA50), and 100 BAWA (BAWA100) of dietary dry matter (DM), were administered to 27 male kids (initial body weight = 17.43 ± kg; age = 105 ± 3.5 days) at random (9/treatment). Bakery waste was included in diets BAWA50 and BAWA100, replacing some of the barley grain. The study lasted for 70 days (7 days used for adaptation and 63 days used for data collection). Kids in the BAWA100 group consumed considerably more (*p* < 0.01) DM, crude protein (CP), and metabolizable energy than those in the BAWA50 and CON groups. However, intakes of neutral detergent fiber (NDF), acid detergent fiber (ADF), and ether extract (EE) were similar (*p* ≥ 0.08) among the treatment diets. The kids’ initial and final body weights, total growth, and average daily weight were not substantially impacted (*p* ≥ 0.13) by the addition of BAWA. All diets had similar feed efficiency when it came to converting feed into body weight increases (*p* ≥ 0.11). Nevertheless, the BAWA diet yielded a considerably lower cost gain (*p* = 0.04) than the CON diet. The three treatment groups had no differences (*p* ≥ 0.15) in DM, ADF, NDF, and EE digestibility. However, compared to the CON group, the digestibility of CP tended to improve (*p* = 0.07) in the BAWA100 and BAWA50 groups. The three experimental groups’ nitrogen (N) intakes did not differ significantly from one another (*p* > 0.33). Additionally, the three diets’ N retention percentage and grams/d were equivalent (*p* > 0.33). Several carcass metrics, such as dressing percentage, hot and cold carcass weights, fasting live weight, and non-carcass components, were not significantly affected (*p* > 0.23) by the addition of BAWA. Additionally, no differences in carcass cut weights were found. The inclusion of BAWA100 and BAWA50 increased loin cut weight (*p* = 0.043) and total lean muscle weight (*p* = 0.001) compared to the CON group. Other components, such as intermuscular fat, subcutaneous fat, total fat, total bone, meat-to-bone ratio, and meat-to-fat ratio, were similar among the three diet groups. Concerning carcass linear dimension measurements, only rib fat depth was greater (*p* = 0.008) in kids fed the BAWA100 diet compared to the BAWA50 and CON diets. No significant variation was observed among the treatments concerning various physicochemical properties of the *longissimus dorsi* muscle in the black goat kids. All measured serum metabolites and hematological parameters were not affected by the incorporation of BAWA into the diet. In summary, the inclusion of 50 and 100 g/kg BAWA in the diet had comparable effects on the black goat kids’ feed intake, digestibility, growth performance, carcass characteristics, meat quality, and overall health. Importantly, feed costs can be reduced by using up to 100 g/kg of BAWA on a DM basis.

## Simple Summary

The goal of raising livestock is to produce animal products of high quality and quantity for human consumption. However, due to the increase in population and demand for these products, as well as the increase in the prices of feed ingredients, livestock producers are facing pressure to maintain the same productivity. In an attempt to solve these problems, researchers have started to include unconventional (i.e., alternative) feeds to diminish the cost of livestock diets and increase profitability. One such candidate is bakery waste. This study was conducted to evaluate the effect of including bakery waste in the diets of growing black goats on growth performance, carcass quality, and blood health status.

## 1. Introduction

An estimated one-third of the world’s food is lost or wasted each year, and these events have detrimental repercussions on society, the economy, and the environment [1]. Approximately 35% of Jordan’s wheat crop is lost or squandered, costing the nation USD 105 million a year [2]. According to Ominski et al. [3], livestock animals possess the ability to upcycle dietary scraps into premium protein sources, including meat, eggs, and milk. A key component of the answer to reducing food waste is feeding waste to animals, which also helps with resource conservation, waste management, food security, pollution control, and climate change mitigation [4].

In light of the deficiency of energy sources in animals’ diets and the increase and fluctuations in the prices of energy feed sources, such as cereal grains, finding alternative low-cost feed is an important strategy to improve the sustainability of the livestock production system and ensure food security [5]. In Jordan, ruminant production currently encounters many challenges, such as climate change, which results in a shortage of feed and rangelands and escalating feed prices [6]. Therefore, more sustainable feed production is needed, along with the exploitation of alternative feed sources.

One of the potential alternative feed sources that could deal with this lack, as well as reducing the high energy costs of animals’ diet (e.g., cereal grain) through reducing the importation of such ingredients, is bakery waste (BAWA), which is characterized as a high concentration of non-fibrous carbohydrates and fat [7]. According to Haddad et al. [8], barley grain is the most common grain used in the Middle East to feed lambs and kids, accounting for between 50% and 75% of dietary dry matter (DM). Compared to barley grain, bread by-products are substantially less expensive (20–40% of the barley grain price) and have a similar composition [9]. In addition to waste resulting from breaking, overcooking, or undercooking during processing, BAWA is a high-energy by-product that is typically found in dry form in bakeries and supermarkets. Examples of this waste include cake leftovers, pieces of bread, croissants, biscuits, dough, and non-marketed products that have passed their expiration date [10]. These are not used for human consumption due to preferences and acceptance by consumers and business policy [11].

In Jordan, numerous bakeries are scattered all over the country. BAWA is an abundant by-product and can be used as a source of feed for sheep and goats [12]. Using BAWA as an energy-rich by-product in livestock nutrition can help recycle wasted food and reduce food competition between livestock and humans. In terms of nutrition, bran cereals have a slightly higher energy content than starchy grains, and their energy precursor profile differs [13]. For instance, compared to native cereal grains, bakery by-products have less starch but more fat, sugar, and fiber [11].

Bakery waste varies greatly in its chemical and ingredient composition owing to its sources, the products it derives from (including the ingredients’ fat content), and regional variability [7]. Many previous studies have shown that the chemical content of the BAWA used in animal feed varies greatly [7,14]. The chemical alterations that take place during baking are another factor that must be taken into account when feeding BAWA to ruminants. According to Drouillard [15], grinding and heating cereal grain may enhance ruminal starch degradation, which may have an impact on feed intake, rumen, and metabolic health. For this reason, the degree of processing that occurs during baking may affect ruminant nutrition. The utilization of starch can be significantly improved by processing grains properly. However, the type of ruminant, source of grain, and processing technique all influence the degree of improvement [16].

Several studies were conducted in which ruminant animals were fed varying amounts of bread by-products or BAWA. In a growing–finishing beef feedlot diet, bread by-products can replace up to 750 g/kg of whole-shelled maize without lowering feedlot performance or meat quality [17]. According to Haddad and Ereifej [12], substituting b 200 g/kg of the diet’s barley DM with bread did not affect kids’ nutritional intake, growth, or digestibility, while lowering feed costs. Furthermore, Afzalzadeh et al. [18] found that up to 250 g/kg of the diets of fattening lambs could contain BAWA without hurting their performance, leading to an enhancement in the quality of the internal and tail fat. Salama et al. [19] suggested substituting dried bakery by-products for yellow maize in lamb-fattening diets at a rate of up to 1000 g/kg for a more cost-effective option.

The potential of BAWA’s inclusion in amounts that could improve the sustainability of animal feed is currently not being fully utilized. This is due, in part, to a paucity of information concerning the influence of replacing barely grains with BAWA on growing black goat kids’ feed intake, growth performance, carcass characteristics, meat quality, and health. Therefore, the study aimed to assess the influence of BAWA on the nutrient intake, growth performance, carcass characteristics, and meat quality of growing black kids. The author hypothesized that the inclusion of up to 50 and 100 g/kg BAWA in the diet would be well tolerated by growing black goat kids, without a negative impact on DM intake and growth performance, and would have an influence on carcass characteristics, meat quality, and health.

## 2. Materials and Methods

### 2.1. Kids and Experimental Diets

This study was carried out in the Faculty of Agriculture’s Animal Field within the Research and Training Department at the Jordan University of Science and Technology (JUST). Before conducting the study, the approval of the Animal Care and Use Committee at JUST was obtained for all procedures of the study. Twenty-seven male kids (initial body weight = 17.43 ± 0.31 kg; age = 105 ± 3.5 days) were randomly allocated into three equal dietary groups (*n* = 9/ diet). The dietary groups varied in terms of the BAWA levels in the diets: kids in the control group (CON) were fed 0 g/kg BAWA, while in the other two dietary groups, the kids were fed either 50 (BAWA50) or 100 (BAWA100) g/kg BAWA on a DM basis, which substituted part of the barley grain. The diets were fed isonitrogenously, contained 160 g/kg of crude protein (CP), and were formulated to match the NRC’s [20] needs for growing kids. Table 1 displays a detailed list of the ingredients, as well as the diets and a BAWA chemical composition analysis. Along with the other ingredients in the diet, the BAWA was obtained from the restaurants of JUST, dried, ground to reduce the size to 1–2 cm in length, and combined. While penned individually (1 m × 1.5 m), the kids were fed every day at about 8:00 and 15:00 and were given seven days to adapt. After acclimation, the feeding trial lasted for 63 days. The kids were provided with free access to food and water during the feeding sessions. After measuring the amount of feed leftovers each day, 10% of it was subsampled, dried for 48 h at 55 °C, and passed through a 1 mm screen, thus readying it for additional examination. To ascertain the daily nutritional intake, samples were placed in nylon bags at −20 °C and left while the DM and other nutrients were analyzed. To reduce variations in the weighing process and to estimate the average daily gain (ADG; final weight − initial weight ÷ number of days), total gain, and feed conversion ratio (FCR; DM intake ÷ ADG gain), at the beginning of the study and every two weeks afterward, the kid’s weights were taken right before feed intake in the morning. The feed and refusal samples were evaluated using AOAC [21] protocols to determine ether extract (EE, determined using the Soxhlet method), CP (using the Kjeldahl process), and DM (at 100 °C in an air-forced oven for 24 h). Additionally, the samples were adapted for use in the ANKOM^2000^ fiber analyzer apparatus (ANKOM Technology Cooperation, Fairport, NY, USA) and analyzed to determine their neutral detergent fiber (NDF) and acid detergent fiber (ADF) contents using the approach described by [22].

After adding varying amounts of BAWA to the kid’s diets, a partial budget analysis was carried out to validate the costs and benefits of each diet. The cost of the diets and the cost of the gains in growing kids fed bakery waste as an alternative feed were calculated in this study. Initially, the cost of each ingredient (per kg), including the bakery waste, was multiplied by the percentage of each ingredient in the diet (Table 1) to calculate the total cost of the diet. Also, other costs, such as water, electricity, and labor, were included in the calculation. To estimate the gain cost, the average weight gain of each kid was multiplied by the FCR. According to the 2024 prices, the cost (USD/1000 kg) of each ingredient used in the study was 380, 775, 365, 700, and 35 for barley grain, soybean meal, wheat straw, additives (minerals and vitamins), and BAWA, respectively. These prices were obtained from the Central Tenders Department at Jordan University of Science and Technology and are comparable to the prices of commercial feed ingredients.

### 2.2. Digestion and Nitrogen Balance Experiment

After 49 days, five kids were randomly selected from each group and moved to individual metabolic cages measuring 1.05 m × 0.80 m to determine nutritional digestibility and nitrogen (N) balance. After completing the adaptation period in the metabolic cages, the kids remained for five days for data collection. The kids’ daily feed intake and leftovers were recorded. Their daily feces were also collected, weighed, and recorded; 10% were retained for additional examination. To assess the N balance (N intake, N loss in feces and urine, retained N (g/d), and N retention (%)), urine was also collected, weighed, and stored in plastic buckets at the same time as fecal collection. The remaining 5% was kept at −20 °C. Each bucket held 50 mL of 6 N HCL to guarantee ammonia retention. The fecal samples obtained from each kid were pooled to form a single sample for each kid. The samples were then placed in an oven at 50 °C to obtain a consistent dehydrated weight. After dehydration, they were ground and passed through a 1 mm screen before being analyzed for DM, NDF, ADF, CP, and EE according to the previously described methods. Urine samples from each kid were pooled to create a single composite sample per kid, which was then analyzed using the Kjeldahl method.

### 2.3. Slaughtering Procedures

At the end of the study, the kids were slaughtered to evaluate the carcass attributes. The kids’ live body weights were measured before slaughter on day 63 of the study and after about 18 h had elapsed since the last feeding (i.e., fasting period). The kids were then transported to the slaughter area at 9:00, where they were humanely slaughtered by trained personnel. The hot carcass weights were recorded immediately after slaughter. The non-carcass components, including the lungs and trachea, heart, spleen, liver, and kidneys, were collected, weighed, and documented. After chilling the carcasses for 24 h at 4 °C, their cold weights were measured and recorded. The dressing percentage was calculated by dividing the cold carcass weight by the live fasting body weight. One day post-slaughter, measurements were taken of the *longissimus dorsi* muscles and chilled carcasses to assess the linear dimensions of fat depth (C), eye muscle depth (B), eye muscle width (A), tissue depth (GR), rib fat depth (J), and eye muscle area. The carcasses were then divided into four parts—rack, leg, shoulder, and loin—which were weighed and recorded. To evaluate meat quality, the *longissimus dorsi* muscles were removed from the loin cuts, vacuum-sealed in plastic bags, and stored in an ultra-low-temperature lab freezer at −20 °C for two weeks.

### 2.4. Meat Quality Measurements

To evaluate meat quality, several parameters were measured, including pH, shear force, cooking loss (CL), water-holding capacity (WHC), and color coordinates (CIE L*, a*, b*), after defrosting. The frozen *longissimus dorsi* muscles were thawed overnight at 4 °C in a refrigerator while still sealed. Each muscle was then sliced into pieces of varying thickness, and each piece’s quality was assessed using different tests. For the color measurement, slices with a thickness of 15 mm were recorded to observe muscle color. The slices were placed on a polystyrene tray, covered with a porous sheet, and allowed to oxygenate for two hours at 4 °C. CL was measured using 25 mm slices. Before cooking, the slices were weighed, placed in plastic bags, and cooked sous vide at 75 °C for 90 min per [23]. After cooking, the slices were weighed again. They were then stored at 4 °C overnight to gather shear force data [24]. Following this, the slices were divided into six smaller cores, each measuring 1 cm³. Shear force in kg was determined using a Warner–Bratzler shear blade with a triangular slot sharp edge (Warner–Bratzler meat shear, GR Manufacturing Co., 1317 Collins LN, Manhattan, KS 66502, USA). WHC was measured using the method presented in [25]. Five grams of raw meat were cut into small pieces, placed between two quartz plates and filter papers, and pressed for five minutes with a 2500 g weight before being removed and weighed. The WHC percentage was calculated as (initial weight − final weight) × 100/initial weight.

### 2.5. Blood Parameters

Blood samples were collected from each kid’s jugular vein using plain vacutainers before feeding at 8:00 on days 1, 30, and 60 of the experiment. The samples were then centrifuged at 1008× *g* for 30 min at 9:00 to separate the serum. The serum was stored in tubes at −20 °C until analysis. The biochemical parameters, including triglycerides, creatinine, low-density lipoprotein (LDL), high-density lipoprotein (HDL), cholesterol, urea nitrogen content, and serum glucose, were measured using a JENWAY 6105 UV/Vis spectrophotometer (Model 6105, Janeway LTD, Felsted, Dunmow ESSEX CM6 3LB, UK). The liver enzyme levels, including aspartate aminotransferase (AST), alkaline phosphatase (ALP), and alanine aminotransferase (ALT), were measured using commercial kits from Biolabo S.A.S., Less Hautes Rivers, Maizy, France.

Using an ABX ABC Vet hematology analyzer (Horiba ABX, Montpellier, France), another blood sample was drawn from the jugular vein at the end of the study (day 63) and placed into vacuum tubes containing EDTA to assess all blood parameters immediately, including white blood cell count (WBC), hemoglobin (Hb), hematocrit (HCT), lymphocytes (LYM), monocytes (MONO), neutrophils (NEO), and eosinophils (EOS).

### 2.6. Statistical Analysis

The data were analyzed by ANOVA 8.1 ed using the MIXED procedure of SAS [26]. In the statistical model, only the explanatory variables (CON, BAWA50, BAWA100) were considered fixed effects. An individual kid was considered an experimental unit in the data analysis. The least-square means were ascertained using relevant pair-wise t-tests in instances when the fixed effects demonstrated significance (*p* ≤ 0.05).

## 3. Results

The nutritional content of the diets was unaffected by the addition of BAWA (Table 1). Nevertheless, the lowest value was associated with the feed cost of the BAWA100 ration (USD441/ton), followed by BAWA50 (USD460/ton) and CON (USD480/ton).

Table 2 displays the impact of BAWA on nutritional intake. The kids in the BAWA100 group consumed considerably more DM, CP, and metabolizable energy (ME; *p* < 0.01) than those in the BAWA50 and CON groups. In contrast to NDF, the ADF and EE intakes were not different among the treatment diets. The kids’ initial and final body weights, total growth, and average daily weight were not substantially impacted by the addition of BAWA (*p* ≥ 0.13). All diets had similar feed efficiency when it came to converting feed into body weight increases (*p* ≥ 0.11). However, the BAWA diet yielded a considerably lower cost gain (*p* < 0.04) than the CON diet.

Table 3 shows the impact of BAWA on the nutritional digestibility and N balance of the young black goats. No significant differences (*p* ≥ 0.15) were observed in the digestibility of DM, ADF, NDF, and EE between the three treatment groups. Nonetheless, compared to the CON group, the BAWA10 and BAWA5 groups’ CP digestibility tended to be better (*p* = 0.07). The N intake of the three experimental groups did not differ significantly (*p* > 0.33). When compared to the CON diet, the kids who were fed the BAWA10 and BAWA5 diets tended to emit less N in their feces (*p* = 0.08). All three experimental groups lost similar amounts of N in their urine (*p* > 0.19). Moreover, N retained as g/d and retention as a percentage (g/100 g) were comparable among the three diets (*p* > 0.33).

Table 4 displays the effects of BAWA on the loin tissues, non-carcass components, and carcass features of the black goat offspring. Several metrics, including dressing percentage, hot carcass weight, cold carcass weight, and fasting live weight, as well as the non-carcass components, such as the heart, liver, spleen, kidney, lungs, and trachea, were not significantly affected (*p* > 0.23) by the addition of BAWA. Furthermore, no significant variations were observed in the weights of the carcass cuts, which included the shoulders, racks, loins, and legs.

Table 4 demonstrates a substantial increase in loin cut weight (*p* = 0.043) and total lean muscle weight (*p* = 0.001) when BAWA of 100 and 50 g/kg was included in comparison to the CON group. The other components were comparable between the three food groups: meat-to-bone ratio, meat-to-fat ratio, total fat, subcutaneous fat, and intermuscular fat.

Except for rib fat depth, the carcass linear dimension measurements were not affected by the treatments (Table 5). Rib fat depth was significantly higher (*p* = 0.008) in the kids fed the BAWA100 diet compared to those fed the BAWA50 and CON diets.

Regarding meat quality characteristics, no significant differences were detected among the treatments regarding the various physicochemical properties of the *longissimus dorsi* muscle in the black goat kids (Table 6).

All measured metabolites that are present in blood serum (i.e., urea N, glucose, cholesterol, LDL, HDL, triglycerides, creatinine, AST, ALP, ALT) were not affected by using BAWA in the diet (Table 7). However, creatinine concentration tended to be higher in the BAWA100 diet compared to the BAWA50 and CON diets.

Additionally, the hematological parameters, including WBC, Hb, HCT, LYM, MONO, NEO, and EOS, were not affected by the inclusion of BAWA in the diets (Table 8).

## 4. Discussion

Considering the feed shortage and the escalating prices of energy feed sources, such as cereal grains, this assessment of the use of alternative feed sources for livestock is substantial regarding the sustainability of animal production. In the present study, no variations were found in the chemical contents of the experimental diets. The similar chemical composition of BAWA and barley resulted in similar CP, NDF, and ADF contents among all the diets, averaging 16.03, 32.29, and 14.66 g/kg, respectively (Table 1). Previous research has revealed that BAWA’s chemical composition varies greatly [7,14]. Sources, products (including the ingredients’ fat content), processing method, and regional variability may all have an impact on variations in BAWA [7,10,27]. Therefore, comparing these values with those found by other researchers is difficult due to this variability. Consequently, before adding BAWA to ruminant diets, its nutritional value should be assessed [10]. According to our study, feed cost and the rate at which BAWA is incorporated into black kid diets have a positive relationship (Table 1). The present findings are consistent with earlier studies that used alternative feeds (e.g., bakery by-products) to decrease the expenses associated with traditional animal nutrition [8,12]. Alternative feeds are less expensive than conventional feeds, which may account for these results [19,28,29,30]. In this study, the use of BAWA100 and BAWA50 diets resulted in a reduction in diet cost by 8.13% and 4.17%, respectively, compared to the CON diet as a result of the reduced percentage of barley grain—an expensive ingredient—to 21% in BAWA100 and to 10% in BAWA50 (Table 1).

The increase in DM intake in the BAWA100 diet compared to the CON diet was reflected in the intake of CP and ME, as presented in Table 2. BAWA is high in digestible carbohydrates (such as starch and sugars) and fats, which can increase the diet’s energy density. The higher DM intake observed in the BAWA100 group in this study indicates that the palatability of the diet is unaffected by the inclusion of 100 g/kg of BAWA. This finding is not consistent with that of Hindiyeh et al. [9], who stated that substituting 100, 200, and 300 g/kg of the barley grain in the diet’s DM with BAWA reduced DM intake, as reflected in the reduced intake of CP, and NDF. However, Haddad and Ereifej [12] showed that replacing up to 200 g/kg of barley grain in the diet DM with by-products did not affect nutrient intake. Franca et al. [10] reported that the inclusion of BAWA in amounts of 250, 500, 750, and 100 g/kg on a DM basis did not affect nutrient intake. Obeidat et al. [31] showed that diets containing 100, 150, and 200 g/kg of bakery-by products, when fed to nursing Awassi ewes, did not affect DM, organic matter, and CP intakes, and NDF and ADF decreased significantly with increasing inclusion levels. Contrary to our results, Afzalzadeh et al. [18] observed no differences in DM intake when lambs were fed diets containing BAWA at levels of 60, 125, and 250 g/kg DM compared to the control diet.

The increase in DM intake was reflected in the final weights, total gain, and ADG weights of the kids fed the experimental diets (Table 4). The final body weights of the kids fed the BAWA50 and BAWA100 diets (25.5 and 25.8 kg, respectively) were higher compared to those of kids fed the CON diet (25.2 kg). On average, the kids fed the BAWA50 and BAWA100 diets gained 14% more weight than the kids fed the CON diets. The growth rates of the kids fed the BAWA50 and BAWA100 diets (130 and 140 g/d, respectively) were also higher compared to kids fed the CON diets (116 g/d). Salama et al. [19] obtained similar effects, improving the live body weight gain of lambs without significant differences, when lambs were fed diets containing bakery by-products at the levels of 125, 250, 375, and 500 g/kg DM compared to a control diet. In the present study, the feed conversion ratio was lower (6.7) for the kids fed the BAWA50 and BAWA100 diets compared with kids that consumed the CON diet (8.6). On average, the body weight gain cost was lower by approximately 27.5% in the BAWA50 and BAWA100 diets compared to the CON diet, as shown in Table 4. The reduction in body weight gain cost may be ascribed to the inclusion of low-cost substitute feed sources—specifically, BAWA—in the diet.

In the current study, no significant variations were found in the experimental diets concerning nutrient digestibility. These findings may be partially explained by the experimental diets’ comparable chemical analyses. In agreement with our results, França et al. [10] found that the inclusion of BAWA at the levels of 250, 500, 750, and 100 g/kg on a DM basis did not influence the nutrient digestibility of sheep. Similarly, Obeidat et al. [31] showed that diets containing 10, 15, and 20% of bakery-by products did not affect nutrient digestibility. Mahmoud [27] stated that the inclusion of bakery by-products at weights of 300 and 600 g/kg on a DM basis did not affect nutrient digestibility and nutritive values, except the digestion coefficients of CP and digestible CP. Furthermore, Haddad and Ereifej [12] demonstrated that increasing the amounts of bakery by-products in goat kids’ rations to 100, 200, and 300 g/kg DM did not affect the digestibility coefficients of DM and NDF. By contrast, Salama et al. [19] reported the highest DM digestibility coefficient for lambs fed rations based on 100 g/kg of bakery by-products and higher digestibility coefficient values for different feed nutrients. They also observed the lowest DM digestibility value when the ration was based on 125 g/kg of bakery by-products.

N intake and N balance were comparable among the treatment groups in the present study. This similarity can be linked to the similarity in the digestibility of CP in the three diets. This result is in agreement with a previous study focusing on a sheep-fed diet containing BAWA at inclusion rates of 250, 500, 750, and 100 g/kg on a DM basis [10]. Salama et al. [32] reported insignificant differences in daily N intake and N retention among different diets when rations containing 0, 150, 300, and 450 g/kg of bakery by-products were mixed with different percentages of yellow corn or grease. Moreover, Salama et al. [19] indicated significant differences in different N balance criteria, except digested N and N balance, among different diet groups containing bakery by-products at levels of 125, 250, 375, and 500 g/kg DM mixed with different percentages of yellow corn.

Assessing the effectiveness of a diet fed to growing kids encompasses two elements: the increase in weight and the change in carcass quality characteristics. Regarding the carcass characteristics, non-carcass components, and loin tissues, the addition of 50 and 100 g/kg of BAWA in the diet led to a significant improvement in loin cut weight and total lean muscle weight compared to the CON group. The improvement in total lean mass may be linked to the increased digestibility of CP in the BAWA10 and BAWA5 diets compared to the CON group. Furthermore, concerning the linear dimension measurements of carcasses, the BAWA100 diet significantly improved rib fat depth in the kids compared to the BAWA50 and CON diets. The consumption of a more energy-dense diet (BAWA100, for example) by animals may lead to more body fat being deposited and an increase in the overall fat-to-lean ratio (Table 4 and Table 5).

In the current study, the homogeneity in DM across the three diets with isonitrogenous qualities was indicative of the kids’ equal growth rates, which led to similar carcass weights and similarities in other attributes. The similar dressing percentages among the three diets may be explained by the kids’ similar slaughter weights. Moreover, the similar effects of the diets on the kids’ non-carcass components and carcass cuts may be ascribed to their similar slaughter and carcass weights. These results are in line with those of Milton and Brandt [33], who reported a significant linear increase in kidney, pelvic, and heart fat and a non-significant increase in the twelfth-rib fat thickness when corn was replaced with dried bakery products (replacing 0, 15, and 30% of the corn), whereas the other carcass characteristics, including hot carcass weight and dressing percentage, were unaffected. Guiroy et al. [17] observed no differences between two diets (based on corn and bread by-products) in terms of their carcass characteristics, including dressing percentage and fat depth. Similarly, Afzalzadeh et al. [18] found no differences in carcass, internal fat, and tail fat weights when lambs were fed diets containing BAWA at levels of 60, 125, and 250 g/kg DM compared to a control diet.

The inclusion of BAWA in the present study did not affect the meat quality characteristics. This finding is consistent with that of Guiroy et al. [17], who reported no significant differences between two diets (based on corn and bread by-products) regarding shear force values, tenderness, juiciness, flavor, and overall acceptability. Our results also agree with those of Grossi et al. [34], who did not report any effect of including former bakery foodstuffs on beef cattle carcass characteristics. Furthermore, the meat quality of broiler chicken was not significantly different among diets containing 0, 100, 200, and 300 g/kg = of bread waste meal [35].

Measurements of the concentrations of blood serum metabolites and hematological parameters are crucial diagnostic tools for animal nutritional status and health conditions, and their concentrations can be influenced by internal and external factors. In this study, the inclusion of BAWA did not affect blood metabolites, hematological characteristics, liver enzymes, and kidney function. As the differences observed following the dietary inclusion of BAWA and the CON diet were not significant, the inclusion of BAWA in the diet was determined not to adversely affect animal health. This finding partially agrees with that of Mahmoud [27], who showed that the inclusion of 300 and 600 g/kg of bakery by-products on a DM basis had no effect on blood parameters, except total proteins, albumin, globulin, and blood glucose.

## 5. Conclusions

This study showed that the partial substitution of barley grain with two levels of BAWA had comparable effects on the digestibility, growth performance, N balance, carcass characteristics, and meat quality of black goat kids. Additionally, the inclusion of BAWA at levels of 50 and 100 g/kg in the diets reduced the feeding cost without having a detrimental effect on the kids’ health. Because of the effects on kid performance, production cost, and health, the author suggests partially substituting BAWA for barley grain in diets as an alternative feed for growing goat kids. Although the use of BAWA did not cause any health problems in the current study, it is recommended that the BAWA is analyzed to determine the nutrient content and mycotoxins.

## Figures and Tables

**Table 1:** Ingredients and chemical compositions of the diets fed to black goat kids.

Item	Diet ^1^		
	CON	BAWA50	BAWA100
**Ingredients (g/kg DM)**			
Barley grain	460	410	365
Soybean meal	220	215	215
Wheat straw	300	300	300
Bakery waste ^2^	0	50	100
Salt	10	10	10
Limestone	9.0	9.0	9.0
Vitamin-mineral premix ^3^	1.0	1.0	1.0
Cost (USD/1000 kg) ^4^	480	460	441
**Nutrients**			
DM, g/kg	912	915	917
CP, g/kg	161	160	160
NDF, g/kg	332	323	313
ADF, g/kg	149	147	144
EE, g/kg	9.1	9.1	9.1
NSC ^5^	39.9	40.8	41.7
ME, Mcal/kg ^6^	2.28	2.31	2.33

^1^ The diets were (1) no bakery waste (CON), (2) 50 g/kg of bakery waste (BAWA50), and (3) 100 g/kg of bakery waste (BAWA100). ^2^ The bakery waste contained 880, 120, 24, 6, 16, and 15 g/kg of DM, CP, NDF, ADF, EE, and sodium, respectively. It also contained 3.2 Mcal/kg of ME. ^3^ The composition per kg was vitamin A, 600,000 IU; vitamin D3, 200,000 IU; vitamin E, 75 mg; vitamin K3, 200 mg; vitamin B1, 100 mg; vitamin B5, 500 mg; lysine, 0.5%; DL–methionine, 0.15%; manganese oxide, 4000 mg; ferrous sulfate, 15,000 mg; zinc oxide, 7000 mg; magnesium oxide, 4000 mg; potassium iodide, 80 mg; sodium selenite, 150 mg; copper sulfate, 100 mg; cobalt phosphate, 50 mg; and dicalcium phosphate, 10,000 mg. ^4^ The values were calculated based on ingredient prices in 2024. ^5^ non-structural carbohydrates. ^6^ The values were calculated based on the tabular value of NRC [20].

**Table 2:** Effects of bakery waste feed on the nutrient intake and growth performance of black goat kids.

Item	Diet ^1^				
	CON (n = 9)	BAWA50 (n = 9)	BAWA100 (n = 9)	SE	*p* Value
**Nutrient intake**					
Dry matter, g/d	897 ^a^	891 ^a^	961 ^b^	16.6	0.010
Crude protein, g/d	145 ^a^	143 ^a^	154 ^b^	2.7	0.012
Neutral detergent fiber, g/d	298	288	301	7.0	0.180
Acid detergent fiber, g/d	134	131	139	2.4	0.076
Ether extract	9	8	8	0.11	0.081
Metabolizable energy, Mcal/d	2.05 ^a^	2.06 ^a^	2.24 ^b^	0.038	0.002
Initial weight, kg	18.0	17.3	17.0	0.31	0.128
Final weight, kg	25.2	25.5	25.8	0.72	0.811
Total gain, kg	7.3	8.2	8.8	0.60	0.160
Average daily gain, g/d	115.8	130.1	139.8	9.59	0.160
FCR ^2^	8.6	6.9	6.5	0.75	0.113
Cost (USD/kg gain)	4.15 ^b^	3.17 ^a^	2.85 ^a^	0.358	0.043

^1^ The diets were (1) no bakery waste (CON), (2) 50 g/kg of bakery waste (BAWA50), and (3) 100 g/kg of bakery waste (BAWA100).^2^ FCR: feed conversion ratio. Different letters indicate a significant difference at *p* < 0.05.

**Table 3:** Effects of bakery waste feed on the nutrients’ in vivo digestibility and the N balance of black goat kids.

Item	Diet ^1^				
	CON (n = 5)	BAWA50 (n = 5)	BAWA100 (n = 5)	SE	*p* Value
**Digestibility coefficients**					
Dry matter	70.58	74.11	72.68	1.610	0.149
Crude protein	79.34	83.45	82.73	1.577	0.067
Neutral detergent fiber	58.36	62.74	59.05	2.099	0.142
Acid detergent fiber	48.39	52.23	50.32	3.972	0.643
Ether extract	72.44	74.77	72.66	2.518	0.611
**N balance**					
N intake, g/d	23.56	22.24	22.61	0.866	0.339
N in feces, g/d	5.14	4.29	4.12	0.391	0.066
N in urine, g/d	7.05	6.14	6.80	0.470	0.195
N retained, g/d	11.37	11.82	11.70	0.973	0.896
Retention, g/100 g	47.97	53.12	51.70	3.371	0.339

^1^ The diets were (1) no bakery waste (CON), (2) 50 g/kg of bakery waste (BAWA50), and (3) 100 g/kg of bakery waste (BAWA100).

**Table 4:** Effects of bakery waste feed on the carcass and non-carcass components, carcass cut weight, and loin cut tissue percentage of black goat kids.

Item	Diet ^1^				
	CON (n = 9)	BAWA50 (n = 9)	BAWA100 (n = 9)	SE	*p* Value
Fasting live weight, kg	24.8	25.0	25.3	1.01	0.831
Hot carcass weight, kg	12.0	11.9	12.4	0.51	0.745
Cold carcass weight, kg	11.4	11.0	11.7	0.252	0.694
Dressing percentage	44.3	45.0	46.4	0.86	0.233
Non-carcass components, kg ^2^	1.31	1.34	1.35	0.048	0.840
Carcass cut weights, kg ^3^	10.8	10.6	11.2	0.50	0.684
Loin weight, g	318.20 ^a^	385.9 ^ab^	412.6 ^b^	27.40	0.043
Intermuscular fat, g	13.9	21.1	24.4	3.42	0.117
Subcutaneous fat, g	17.9	24.7	24.9	3.21	0.247
Total fat, %	9.7	11.5	11.8	1.04	0.311
Total lean, %	41.7 ^a^	49.7 ^b^	48.8 ^b^	1.34	0.001
Total bone, %	33.6	29.2	30.9	2.10	0.343
Meat-to-bone ratio	1.34	1.76	1.64	0.153	0.178
Meat-to-fat ratio	4.48	4.87	4.34	0.480	0.731

^1^ The diets were (1) no bakery waste (CON), (2) 50 g/kg bakery waste (BAWA50), and (3) 100 g/kg bakery waste (BAWA100). ^2^ The non-carcass components included the heart, liver, spleen, kidney, lungs, and trachea. ^3^ The carcass cuts included the shoulder, racks, loins, and legs. Different letters indicate a significant difference at *p* < 0.05.

**Table 5:** Effects of bakery waste feed on the linear dimensions of the *longissimus dorsi* muscle in black goat kids.

Item	Diet ^1^				
	CON (n = 9)	BAWA50 (n = 9)	BAWA100 (n = 9)	SE	*p* Value
Leg fat depth (L3), mm	1.31	1.22	1.38	0.186	0.677
Tissue depth (GR), mm	8.00	9.83	9.19	0.610	0.122
Rib fat depth (J), mm	1.32 ^a^	1.61 ^a^	2.219 ^b^	0.196	0.008
Eye muscle area, cm^2^	6.67	6.36	6.72	0.586	0.804
Eye muscle width (A), mm	42.81	44.22	42.63	1.541	0.579
Eye muscle depth (B), mm	18.50	19.44	18.88	1.023	0.718
Fat depth (C), mm	1.00	1.00	1.06	0.032	0.307
Shoulder fat depth (S2), mm	1.02	1.01	1.11	0.042	0.208

^1^ The diets were (1) no bakery waste (CON), (2) 50 g/kg of bakery waste (BAWA50), and (3) 100 g/kg of bakery waste (BAWA100). Different letters indicate a significant difference at *p* < 0.05.

**Table 6:** Effects of bakery waste feed on the physicochemical properties of the *longissimus dorsi* muscle in black goat kids.

Item	Diet ^1^				
	CON (n = 9)	BAWA50 (n = 9)	BAWA100 (n = 9)	SE	*p* Value
pH ^2^	5.88	5.89	5.88	0.008	0.537
Cooking loss, %	43.98	43.33	45.60	0.735	0.107
Water holding capacity, %	32.90	33.54	33.99	1.623	0.866
Shear force, kg/cm ^2^	6.31	7.41	7.56	0.487	0.169
Color coordinates					
L* (whiteness)	37.2	35.0	35.5	1.40	0.415
a* (redness)	2.3	2.2	1.7	0.35	0.483
b* (yellowness)	23.0	23.6	24.7	0.92	0.388

^1^ The diets were (1) no bakery waste (CON), (2) 50 g/kg of bakery waste (BAWA50), and (3) 100 g/kg of bakery waste (BAWA100). ^2^ The pH was measured after thawing.

**Table 7:** Effects of bakery waste feed on blood metabolites in black goat kids.

Item	Diet ^1^				
	CON (n = 9)	BAWA50 (n = 9)	BAWA100 (n = 9)	SEM	*p* Value
Blood urea nitrogen, mg/dL	36.43	34.76	36.43	1.612	0.590
Glucose, mg/dL	54.09	51.09	52.82	1.631	0.283
Cholesterol, mg/dL	74.48	67.28	69.89	2.665	0.175
HDL, mg/dL ^2^	34.07	31.15	32.30	1.245	0.266
LDL, mg/dL ^2^	34.83	32.03	32.81	1.712	0.500
Triglycerides, mg/dL	19.11	21.24	21.15	1.201	0.381
Creatinine, mg/dL	0.92	0.81	1.05	0.0721	0.080
AST, IU/L ^2^	39.74	36.43	35.96	2.419	0.494
ALT, IU/L ^2^	9.93	7.82	9.26	0.988	0.321
ALP, IU/L ^2^	86.48	84.41	67.56	11.720	0.468

^1^ The diets were (1) no bakery waste (CON), (2) 50 g/kg of bakery waste (BAWA50), and (3) 100 g/kg of bakery waste (BAWA100). ^2^ HDL: high-density lipoprotein, LDL: low-density lipoprotein, AST: aspartate aminotransferase, ALT: alanine aminotransferase, ALP: alkaline phosphatase.

**Table 8:** Effects of bakery waste feed on some hematological parameters in black goat kids.

Item	Diet ^1^				
	CON (n = 9)	BAWA50 (n = 9)	BAWA100 (n = 9)	SEM	*p* Value
WBC ^2^, ×10^3^/µL	13.04	15.04	15.91	1.372	0.133
HB ^2^, g/dL	8.38	8.86	8.29	0.268	0.106
HCT ^2^, %	25.56	26.22	25.56	1.118	0.792
LYM ^2^, %	46.44	49.56	49.78	4.994	0.761
MONO ^2^, %	2.11	1.56	1.33	0.764	0.588
NEO ^2^, %	49.33	48.11	48.44	5.322	0.972
EOS ^2^, %	1.44	0.78	0.44	0.527	0.187

^1^ The diets were (1) no bakery waste (CON), (2) 50 g/kg of bakery waste (BAWA50), and (3) 100 g/kg of bakery waste (BAWA100). ^2^ WBC: white blood cells, HB: hemoglobin, HCT: hematocrit, LYM: lymphocyte, MONO: monocytes, NEO: neutrophils, EOS: eosinophils.

## Data Availability

The data will be provided upon request from the author.
